# Return to Play After Hamstring Injuries: A Qualitative Systematic Review of Definitions and Criteria

**DOI:** 10.1007/s40279-015-0468-7

**Published:** 2016-01-14

**Authors:** Nick van der Horst, Sander van de Hoef, Gustaaf Reurink, Bionka Huisstede, Frank Backx

**Affiliations:** Department of Rehabilitation, Nursing Science and Sports, University Medical Center Utrecht, Rudolf Magnus Institute of Neurosciences, P.O. Box 85500, 3508 GA Utrecht, The Netherlands; Department of Orthopaedics, Erasmus Medical Centre, Rotterdam, The Netherlands

## Abstract

**Background:**

More than half of the recurrent hamstring injuries occur within the first month after return-to-play (RTP). Although there are numerous studies on RTP, comparisons are hampered by the numerous definitions of RTP used. Moreover, there is no consensus on the criteria used to determine when a person can start playing again. These criteria need to be critically evaluated, in an attempt to reduce recurrence rates and optimize RTP.

**Objective:**

To carry out a systematic review of the literature on (1) definitions of RTP used in hamstring research and (2) criteria for RTP after hamstring injuries.

**Study Design:**

Systematic review.

**Methods:**

Seven databases (PubMed, EMBASE/MEDLINE, CINAHL, PEDro, Cochrane, SPORTDiscus, Scopus) were searched for articles that provided a definition of, or criteria for, RTP after hamstring injury. There were no limitations on the methodological design or quality of articles. Content analysis was used to record and analyze definitions and criteria for RTP after hamstring injury.

**Results:**

Twenty-five papers fulfilled inclusion criteria, of which 13 provided a definition of RTP and 23 described criteria to support the RTP decision. “Reaching the athlete’s pre-injury level” and “being able to perform full sport activities” were the primary content categories used to define RTP. “Absence of pain”, “similar strength”, “similar flexibility”, “medical staff clearance”, and “functional performance” were core themes to describe criteria to support the RTP decision after hamstring injury.

**Conclusion:**

Only half of the included studies provided some definition of RTP after hamstring injury, of which reaching the athlete’s pre-injury level and being able to perform full sport activities were the most important. A wide variety of criteria are used to support the RTP decision, none of which have been validated. More research is needed to reach a consensus on the definition of RTP and to provide validated RTP criteria to facilitate hamstring injury management and reduce hamstring injury recurrence.

PROSPERO systematic review registration number: CRD42015016510.

**Electronic supplementary material:**

The online version of this article (doi:10.1007/s40279-015-0468-7) contains supplementary material, which is available to authorized users.

## Key Points

**Table:** 

There is no consensus within literature on how return-to-play after hamstring injury should be defined.
Return-to-play decision making after hamstring injury lacks standardization and clear criteria.

## Introduction

“When will I be able to play again?” This question about return-to-play (RTP) in sports is of great importance for every athlete after a hamstring injury. The major concern of athletes, trainers, management, and other stakeholders is to start playing as soon as possible, but this might be in conflict with the athlete’s actual physical fitness and readiness for match play [1–3]. This is emphasized by the high rate of recurrence of hamstring injuries (12–33 %) [4–7]. This high rate of recurrence is suggested to occur because of inadequate rehabilitation and/or too early RTP [8, 9]. Of these recurrences, 59 % occur within the first month after RTP [10]. Recurrent hamstring injuries require more extensive rehabilitation than the initial injury, and a previous injury is the undisputed single risk factor for future injury [11, 12]. These hamstring injury rates have not improved over the last 20–30 years in professional soccer and Australian Football [13–15].

Although there have been numerous studies of RTP after hamstring injuries in recent years, the actual term is seldom explicitly defined, with definitions such as “return to sport”, “return to competition”, “return to competitive play”, “return to pre-injury level”, and “return to activity” being used [16–19]. Studies on RTP after other musculoskeletal injuries such as anterior cruciate ligament injury and ankle injury, are also hampered by the lack of a clear definition for RTP [20–22]. This makes a comparison of study outcomes difficult and emphasizes the need for a clear definition of RTP.

In addition to the lack of a clear definition of RTP, there is no consensus in the literature or among sports medical practitioners on when an athlete is ready to resume playing after a hamstring injury. In the absence of clear scientific evidence, RTP decisions are not standardized [23, 24], and this has prompted interest in criteria to support the RTP decision after hamstring injury [25, 26]. These criteria need to be critically evaluated to reduce recurrence rates and optimize RTP.

The aim of this study was therefore to carry out a systematic review of the literature on (1) definitions of RTP used in hamstring research and (2) criteria for RTP after hamstring injuries.

## Methods

### Study Design

A systematic search was conducted in PubMed, EMBASE/MEDLINE, CINAHL, PEDro, Cochrane, SPORTDiscus, and Scopus to collect articles describing a definition or criteria for RTP. This review adheres to the Preferred Reporting Items for Systematic Reviews and Meta-Analyses (PRISMA) Guidelines [27]. Registration in the PROSPERO international database of prospectively registered systematic reviews was performed prior to study initiation (registration number CRD42015016510) [28].

### Search Strategy

The search strategies, containing key words such as “return to play”, “return to sport”, and “hamstring injury”, were developed by the primary author (NH) in collaboration with a specialized librarian (see Electronic Supplementary Material Appendix S1). Searches were undertaken from the date of database inception to November 2014. The same databases were then searched independently by two authors (NH, SH). Cohen’s Kappa was calculated for interobserver agreement. All references of the included studies were assessed for inclusion if missed by the initial search.

### Eligibility Criteria

Retrieved articles were screened by two independent authors (NH, SH). Article selection was not limited by study design. Studies needed to describe a definition of, or criteria for, RTP after acute hamstring injury in adult athletes (aged >18 years). Articles that used definitions adopted from other studies were excluded, as were studies that reported only on RTP after surgical interventions. Additionally, articles not available as full text were excluded, although corresponding authors were contacted for information. Differences in article selection and inclusion between the two researchers were resolved in a consensus meeting or, if necessary, a third author (BH) was consulted to make the final decision.

### Data Extraction

If multiple articles were published by the same research group and used the same definition and/or criteria, data were extracted from only one of the articles. The following data were extracted using standardized extraction forms by two authors (NH, SH): first author and year of publication; population and study design; definition of hamstring injury; definition of RTP; described criteria for RTP (Table 1).

**Table 1:** Definition of RTP and criteria for RTP after hamstring injury within the included studies, including step 1 of content analysis

Reference	Study design	Study population, sex, age in years (mean, SD)	Definition of hamstring injury	Definition of RTP after hamstring injury^a^	Criteria for RTP after hamstring injury^a^
Hamid et al. [36]	RCT	Patients; N/R; age >18 years	Grade-2 hamstring muscle injury	Full activities with progressive increase of training load until reaching pre-injury level	Pain free on direct palpation Pain free on hamstring contraction Pain free on active knee extension test Symmetrical range of movement with unaffected side (difference between affected and unaffected side of <10°) Concentric hamstring strength (60°/s, 180°/s, and 300°/s) within 10 % of uninjured side
Askling et al. [37]	Prospective cohort study	18 Sprinters; 8 F: 10 M; 15–28 years and 15 Dancers; 1 M: 14 F; 16–24 years	First time acute sudden pain from the posterior thigh when training, competing, or performing	Able to train, compete, or perform at pre-injury level	Sprinters: competing at similar best times as pre-injury level Dancers: being able to train and perform without restriction
Askling et al. [38]	Cohort study	11 Healthy students; 5 M: 6 F; age 28 ± 7 years and 11 athletes; 8 M: 3F; age 21 ± 7 years	Unilateral, MRI-verified acute hamstring strain	No signs of remaining injury on clinical examination of the injured leg	No pain during palpation and strength testing No strength difference between legs Range of motion during passive straight leg raise should be close (<10 % deficit) to that of the uninjured leg No pain from static contraction in the end position of straight leg raise
Connell et al. [39]	Prospective cohort study	61 M professional Australian Football players; age 24 ± 3.8 years	Acute onset of posterior thigh pain or stiffness, disabling the player from training or match play	Return to competition (completed game)	None provided
Coole and Gieck [40]	Clinical commentary	N/A	Not provided	Not provided	Isokinetic testing within 10 % of normal: equal flexibility Pain-free 2-mile endurance run Pain-free controlled sprinting Pain-free functional activities peculiar to sport Full return of cerebromuscular capabilities
Cooper and Conway [41]	Case series	25 Athletes; N/R; N/R	Complete distal semitendinosus tendon ruptures	Play at the preinjury level or, for those athletes whose sport was not in season, clearance to play	Return of 80 % isotonic knee flexion strength as compared with the normal opposite leg No pain when sprinting Having progressed through a sport-specific functional rehabilitation program Being cleared to play at the preinjury level of professional or amateur competition
Delveaux et al. [42]	Survey report	N/A	Not provided	Not provided	Complete pain relief Muscle strength performance Subjective feeling reported by player Muscle flexibility Specific soccer test performance Respect of a theoretical period of competition break Running analysis Physical fitness Balance control assessment Medical imaging Dynamic functional testing performance Correction of potential sacroiliac or lumbar joint dysfunction Quadriceps: hamstrings EMG analysis
Dembowski et al. [43]	Case report	1 M collegiate pole vaulter; age 18 years	Not provided	Not provided	Eccentric strength within 10 % of the uninvolved extremity Single leg triple hop within 10 % bilaterally Pain free Illinois Agility Test within 18.4 s
Fuller and Walker [33]	Prospective cohort study	55 M professional football players; N/R	Any injury that prevented a player from taking a full part in training activities typically planned for the day and/or match play, not including the day on which the injury was sustained	Achievement of a 100 % recovery score on fitness and skill testing	Pain-free completion of match pace football element assessment at normal match speed
Hallén and Ekstrand [44]	Cohort study	89 M professional football teams; N/R	A traumatic distraction or overuse thigh muscle injury to the anterior or posterior thigh muscle groups leading to a player being unable to fully participate in training or match play	The decision-making process of returning an injured or ill athlete to practice or competition. This ultimately leads to medical clearance of an athlete for full participation in sports	Not provided
Heiderscheit et al. [45]	Clinical commentary	N/A	Not provided	Not provided	Four consecutive pain-free repetitions of maximum effort manual strength test in each prone knee flexion position (90° and 15°) Less than a 5 % bilateral deficit should exist in the ratio of eccentric hamstring strength (30°/s) to concentric quadriceps strength (240°/s) Knee flexion angle at which peak concentric knee flexion torque occurs should be similar between limbs Functional ability testing (sport-related movements specific to the athlete, with intensity and speed near maximum)
Heiser et al. [46]	Retrospective cohort study	Football players; N/R; N/R	A sudden pain in the posterior thigh during a movement requiring rapid contraction of the hamstring muscles	Not provided	Run at “near-full” speed Display of adequate agility Strength at 95 % of baseline score Hamstring:quadriceps ratio of 0.55 or greater at a testing speed of 60°/s
Kilcoyne et al. [47]	Retrospective case series	48 Athletes; 40 M: 8 F; age 18–20 years, *n* = 30 age 21–25 years, *n* = 17	Sudden posterior thigh pain while running or jumping, physical disability, pain with resisted prone knee flexion, and tenderness to palpation of the muscle-tendon unit of the hamstring	Not provided	Ability to perform at 90 % speed during full-sprint drills Athletes’ self-perceiving equivalent hamstring function and strength between injured and uninjured legs on strength testing Pain-free during all drills, including rolling sprints
Malliaropoulos et al. [48]	Cohort study	260 Elite track and field athletes; 150 M: 110 F; age 18–25 years	Acute, first-time posterior thigh muscle injury sustained during training or competition	Training or competing at preinjury level without any symptoms or signs of injury (such as pain, swelling, and/or tenderness)	Normalization of AROM deficit Isokinetic hamstring strength deficit of less than 5 % measured at 60°/s and 180°/s compared with the injured side No difference in single-leg triple-hop test
Mendiguchia and Brughelli [16]^b^	Clinical commentary	N/A	Not provided	Not provided	Optimum angle for peak torque <28° during knee flexion Optimum angle for peak torque <8° symmetry between legs Similar hip extension strength (<10% asymmetry) Similar horizontal force between legs (<20 % asymmetry) Edema size and/or length as shown on MRI Lumbar rotation stability (no anterior pelvic tilt during ASLR test)
Moen et al. [49]	Prospective cohort study	80 Competitive or recreational athletes; N/R; 29 ± 7 years	Acute, MRI-verified, posterior thigh pain	Return to unrestricted sports activity in training and/or match play	Clearance by supervising physiotherapist
Nett et al. [50]	Conference abstract	24 Athletes; 19 M: 5 F; age 24 years (range 16–46 years)	Acute clinical grade 1–2 hamstring injuries	Not provided	Full hamstring strength No tenderness No pain No side-to-side differences during running
Orchard [51]	Clinical commentary	N/A	Not provided	Not provided	Normal strength (>90 % of the unaffected side) Normal range of motion Performance at training dictates readiness for matches
Petersen and Hölmich [52]	Clinical commentary	N/A	An incident occurring during scheduled games/competitions or practice and causing the athlete to miss the next game/competition or practice session	Not provided	Pain-free participation in sport-specific activities
Petersen et al. [53]	Case series	942 Soccer players; N/R; N/R	Sudden physical complaint of posterior thigh sustained during a soccer match or training, irrespective of medical attention or time loss from soccer activities	Availability for match selection or full participation in team training if the injury occurred during a period without match play	Consultation between medical staff and player
Reurink et al. [26]	Cohort study	53 M athletes; mean age 27 years (range 18–46 years)	Clinical diagnosis of hamstring injury by registered sports medicine physician	Successful and asymptomatic completion of physiotherapy program, including functional sport-specific activities	Successful and asymptomatic completion of a functional criteria-based, four-staged physiotherapy program, including a final supervised sport-specific (outdoor) training phase Less than 10 % side-to side-difference at isokinetic strength testing 5 days of team training before participation on partial match play
Sanfilippo et al. [54]	Prospective cohort study	25 Recreational athletes; 20 M: 5 F; 24 ± 9 years	Acute sudden-onset hamstring injury	Not provided	No significant pain with straight leg raise Full hamstring strength No tenderness to palpation No apprehension during full-effort, sport-specific movements Clearance by physiotherapist
Silder et al. [55]	RCT	24 Athletes; 19 M: 5 F; age 24 ± 9 years	Sudden-onset posterior thigh pain	Completion of rehabilitation	No palpable tenderness along the posterior thigh Subjective readiness (no apprehension) after completing a series of progressive sprints working up to full speed 5/5 on manual muscle testing
Tol et al. [25]^b^	Cohort study	52 M players; mean age 24 years (range 18–38 years)	MRI-positive hamstring injury	Not specified	Painless passing and running Painless shooting scenarios Painless competitive 1 vs 1 drills Painless scoring scenarios
De Vos et al. [56]	Prospective cohort study	64 Patients; 61 M: 3 F; median age 28 years (range 23–33 years)	Clinical and radiological diagnosis of grade 1 or 2 acute hamstring injury	Completion of criteria-based rehabilitation program	Symptom free (e.g., pain and stiffness) during: full range of motion full-speed sprinting sport-specific movements (such as jumping and cutting) Clearance by physical therapist Unhindered functional sport-specific testing

*AROM* active range of motion, *ASLR* active straight leg raise, *EMG* electromyography, *F* female, *M* male, *MRI* magnetic resonance imaging, *N/A* not applicable, *N/R* not reported, *RCT* randomised controlled trial, *RTP* return-to-play, *SD* standard deviation

^a^Step 1 of content analysis: results of open coding

^b^These studies used different criteria at different stages in the rehabilitation program; only criteria that supported the final RTP decision were included in this table

### Data Analyses

The methodological quality of the included articles was not assessed because the aim of this systematic review was to collate and synthesize all information on the definition of RTP and its criteria. Descriptive statistics were used to summarize the frequency of different study designs. Definitions of, and criteria for, RTP were analyzed by content analysis [29, 30]. Two authors (NH, SH) separately performed each step of the analytical process to ensure adequate categorization of information and appropriate thematic analysis consistent with the literature [29]. After each step, coding procedures were discussed and if no consensus was reached, a third author (BH) made the final decision.

### Content Analysis

The first step in the content analysis was to create tentative labels for RTP definition and criteria within the articles, using an open coding procedure [31]. Open coding means that notes and headings are written in the text while it is read. The written material is read through again, and as many headings as necessary are written down in the margins to describe all aspects of the definition and criteria for RTP [32].

The second step was to perform axial coding to identify relationships among open codes. Axial coding, termed “axial” because coding occurs around the axis of a category, links categories at the level of properties and dimensions [31]. Two authors (NH, SH) independently assessed whether headings identified during open coding were associated [30]. For instance, one article might describe concentric hamstring strength testing and no findings on magnetic resonance imaging (MRI) as criteria to support the decision for RTP after hamstring injury. A second article might describe eccentric hamstring strength testing as a criterion. A relationship between eccentric and concentric strength testing could be identified from these codes (e.g., “strength testing”), whereas the relationship between no findings on MRI and eccentric hamstring strength testing is more far-fetched.

In the third step, final content categories were identified by selective coding [31]. In this phase, content categories are established and it is determined whether axial coding categories are correlated with these content categories (such as a hypothetical content category “strength testing” as stated in the aforementioned example) [31].

## Results

### Search Results

**Fig. 1:**
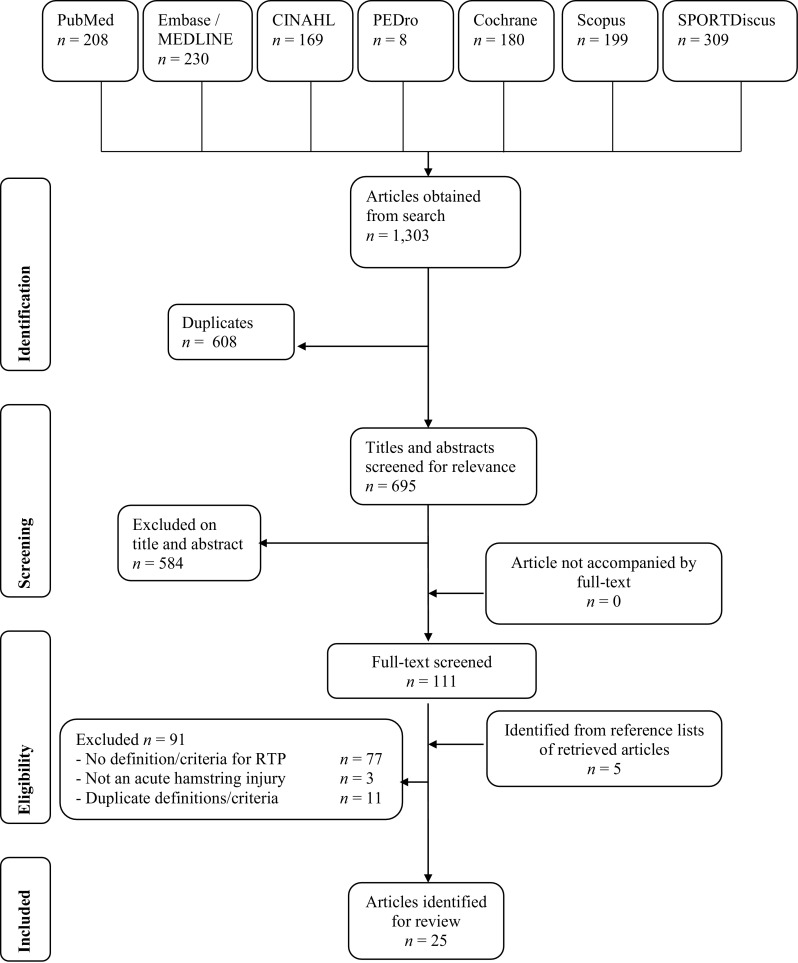
Study selection flow chart

Of 1303 articles retrieved, 608 were excluded as duplicate publications and a further 584 were excluded after screening of the title and abstract (Fig. 1). The remaining full-text articles (*n* = 111) were checked for relevant content, based on eligibility criteria, by two researchers (NH and SH). Five articles were identified from the reference lists of retrieved articles. Our third author (BH) was consulted to decide on two articles for potential inclusion. The article by Fuller et al. [33] was included and one other article was excluded [34]. In total, 25 articles met the inclusion criteria. Cohen’s Kappa was 0.79 at this point, indicating substantial agreement [35].

### Types of Publications and Their Contents

Of the 25 articles, 18 were clinical studies (2 randomized controlled trials, 12 cohort studies, 3 case series, and 1 case report), 1 a narrative review, 4 clinical commentaries, 1 a survey report, and 1 a conference abstract (Table 1).

### Definition of RTP

Thirteen articles (52 %) defined RTP (Table 1).

#### Coding

**Fig. 2:**
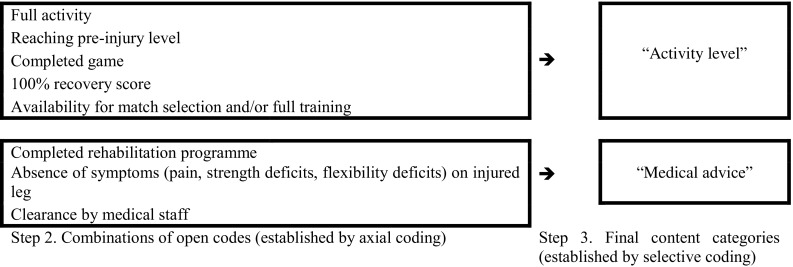
Axial and selective coding of definition for return-to-play, steps 2 and 3 of content analysis

Open coding of the relevant content of the articles resulted in open codes for the “definition of RTP after hamstring injury” (Table 1, “definition of RTP”). After axial coding, related codes were grouped into two final content categories (e.g., selective coding): “activity level” and “medical advice” (Fig. 2).

#### Activity Level

Most authors used terms such as “reaching pre-injury level” [36, 37, 41, 48] and “full activity” [36, 44, 49, 53] to define RTP after hamstring injury. Other terms include “availability for match selection and/or full training” [41, 49, 53], “a completed game” [39], and “a 100 % recovery score on fitness and skill testing” [33].

#### Medical Advice

RTP after hamstring injury was also defined on the basis of medical information [26, 38, 40, 44, 48, 55, 56]. “Absence of symptoms on injured leg” [38, 48], “clearance by medical staff” [41, 44, 56], and “completion of a rehabilitation program” were used as terms to define RTP [26, 55, 56]. Most articles provided additional medical criteria to support the RTP definition [26, 38, 41, 48, 55, 56] (see Sect. 3.4).

### RTP Criteria

Of the 25 included articles, 23 articles (92 %) provided criteria for RTP after a hamstring injury (Table 1).

#### Coding

**Fig. 3:**
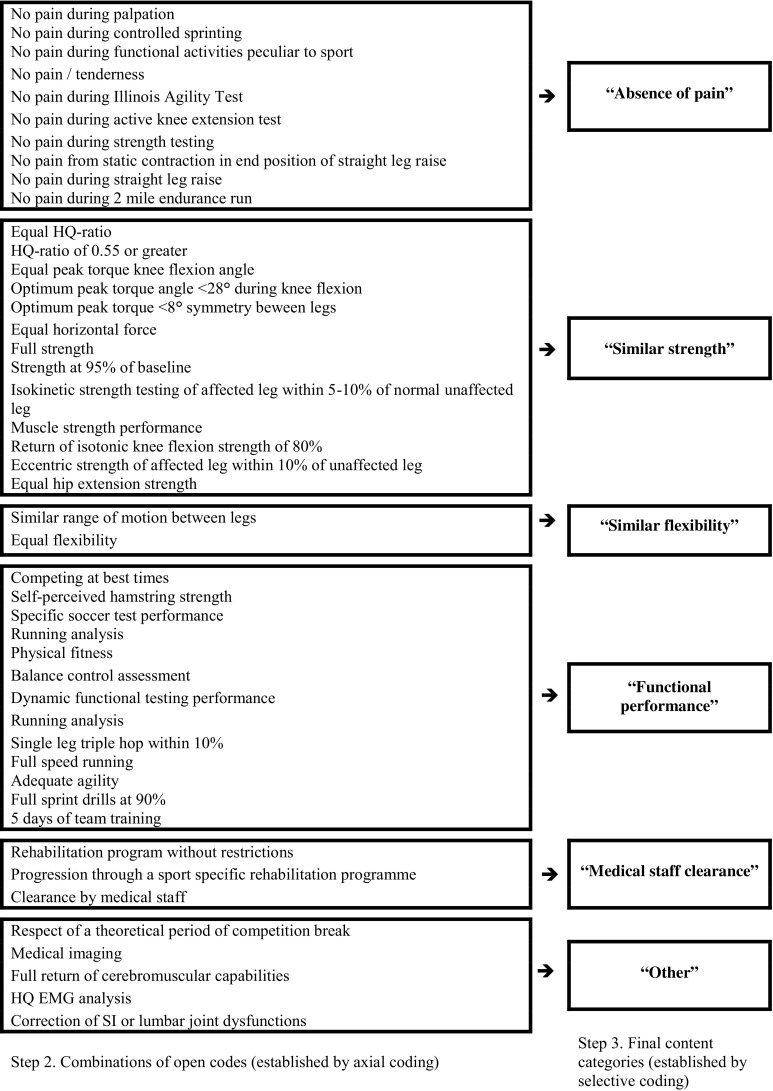
Axial and selective coding of criteria for RTP, steps 2 and 3 of content analysis. *EMG* electromyography, *HQ* hamstrings–quadriceps, *RTP* return-to-play, *SI* sacroiliac

After open coding and subsequent axial coding of criteria for RTP (Table 1, “criteria for RTP after hamstring injury”), related codes were grouped into five final content categories (e.g., selective coding): “absence of pain”, “similar strength”, “similar flexibility”, “medical staff clearance”, and “functional performance” (Fig. 3).

#### Absence of Pain

Absence of pain on palpation and during performance testing was used as a criterion for RTP after hamstring injury in 15 studies [25, 26, 33, 36, 38, 40–43, 45, 47, 50, 52, 54–56]. In some studies, pain was tested via direct palpation of the hamstring muscle [36, 37, 54, 55]. Askling et al. and Hamid et al. additionally stated that hamstring contraction should not elicit pain when tested in the end position of the passive straight leg raise [36, 37]. Other studies considered a pain-free state during strength and flexibility testing as fitness for RTP, but did not mention how strength and flexibility tests were performed [37, 45, 54, 56]. Pain-free running, such as in a 2-mile endurance run or controlled sprinting, and pain-free functional activities peculiar to a given sport were also used as criteria for RTP [25, 33, 40, 41, 45, 47, 50, 52, 54, 56].

#### Similar Strength

A similar hamstring strength in the affected and the unaffected legs was used as a criterion in 15 studies [16, 26, 36, 38, 40–43, 45–48, 50, 51, 54, 55]. Most studies considered a deficit of <10 % as being similar [16, 26, 36, 40, 43, 45, 46, 48, 54].

Hamstring strength was measured in different positions with different tools. Kilcoyne et al. assessed strength as athletes’ self-reported hamstring function during strength testing [47]. Other studies reported manual resistance testing at the heel with the knee flexed at 0°, 15°, 45° and 90° in prone position [38, 45]. There were also variations in test procedures with the tibia in the neutral, external rotated, and internal rotated positions [55]. Dembowski et al. measured eccentric hamstring strength with a hand-held dynamometer using the break method [43]. Mendiguchia tested isokinetic hip extension at 60°/s [16], where other included studies tested at 60°/s, 180°/s, 240°/s, and 300°/s [25, 36, 40]. Cooper also assessed isotonic knee flexion strength, but differed from other studies as the criterion for RTP required the injured leg to reach 80 % strength, instead of >90 % strength, relative to the normal opposite leg [41]. Multiple studies endorsed isokinetic strength testing under both concentric and eccentric conditions, stating that there should be less than a 5–10 % deficit in the ratio of eccentric hamstring strength (30°/s, 60°s, or 180°/s) to concentric quadriceps strength (240°/s) between the injured and uninjured legs [36, 45, 46, 48, 54]. Heiser et al. stated the hamstring:quadriceps ratio should be ≥0.55 at a testing speed of 60°/s [46]. In addition, it was suggested that the knee flexion angle at which peak concentric knee flexion torque occurs should be similar between limbs [16, 45].

#### Similar Flexibility

Normal hamstring flexibility or range of motion was used as a criterion in seven studies [36, 38, 40, 42, 45, 48, 51]. Only the study by Askling et al. specified normal hamstring flexibility as a <10 % deficit between the injured and the uninjured legs [38].

Flexibility or range of motion was tested via passive straight leg raise [38] or by active knee extension in the supine position with the hip flexed at 90° [48]. Other studies did not specify measurement methods or cut-off values for flexibility measurements.

#### Functional Performance

Thirteen studies reported performance during field testing as a criterion for RTP after hamstring injury [25, 26, 37, 42, 43, 45–48, 50, 51, 53, 56]. One study used best sprint times comparable to those before injury [37]. Nett et al. stated that no asymmetry should occur during running [50], whereas Reurink et al. stated no asymmetry should be present during the sport-specific (outdoor) training phase [26], although neither study defined asymmetry. Training and performance without any restriction was also reported as a criterion [25, 37, 56]. According to Heiderscheit et al., functional ability testing should incorporate sport-related movements performed at near-maximum intensity and speed [45]. Tol et al. specified this further by using pain-free running, passing, shooting, scoring, and competitive one-to-one drills as criteria for RTP for soccer players [25]. Single-leg triple hops and a pain-free Illinois Agility Test within 18.4 s were also reported as functional performance criteria for RTP after hamstring injury [43, 48]. Reurink et al. additionally stated that, after full recovery, 5 days of team training are required before clearance for (partial) match play [26].

#### Medical Staff Clearance

Five studies reported that the athlete should be certified as medically fit before returning to play [41, 49, 53, 54, 56], but few studies described how this was done. In the study by Petersen et al., this decision was made in consultation between medical staff and the player [53]. Cooper et al. mentioned additional criteria (e.g., return of >80 % isotonic knee flexion strength as compared with the normal opposite leg, no pain when sprinting, and having progressed through a sport-specific rehabilitation program) that need to be met before medical staff give their approval for RTP [41]. Three studies reported that the athlete should have progressed through a sport-specific rehabilitation program without restrictions before RTP [26, 41, 56], but none of the studies described the content of such a program.

#### Other

Other criteria for RTP after hamstring injury used were full return of cerebromuscular capabilities (not further specified by Coole et al.), extent of edema, and lumbar rotation stability [16, 40]. Anterior pelvic tilt was not allowed during the active straight leg raise test in the study by Mendiguchia and Brughelli [16]. Additionally, in the study by Delvaux et al., sports physicians reported adherence to a theoretical period of competition break, medical imaging, correction of sacroiliac or lumbar dysfunction, and quadriceps-hamstrings electromyography analysis as criteria for RTP [42].

## Discussion

### Statement of Principal Findings

In this article, we systematically reviewed the literature on definitions and criteria for RTP after hamstring injuries. Only 52 % of the included articles defined RTP, whereas 92 % provided criteria to support the RTP decision. Although different definitions have been used, we found that terms referring to “activity level” (e.g., reaching pre-injury level, full activity) or “medical advice” (e.g., clearance by medical staff, absence of symptoms, and completion of a rehabilitation program) were often used to define RTP after hamstring injury.

A variety of criteria have been used to support the RTP decision, subdivided into five content categories: “absence of pain” (e.g., on palpation and during performance), “similar strength” (e.g., a <10 % deficit between the affected and unaffected leg), “similar flexibility”, “medical staff clearance”, and “functional performance”.

### Strengths of the Study

Various medical and sport databases were used to collect detailed information on the definition of RTP after acute hamstring injury [57], and the inclusion of studies using a different methodology provides a broad understanding of RTP. PRISMA guidelines were followed as much as possible to ensure transparent reporting of this systematic review [27].

Article selection and data retrieval were done by two researchers independently, to maximize the inclusion of relevant articles and data [58]. The third author was consulted twice to decide on the inclusion of two articles, but this did not significantly affect our study results. We used content analysis to systematically identify and synthesize recurring themes within the definitions of RTP after acute hamstring injury [29, 30].

### Limitations of the Study

No search limits were placed on level of evidence, as is common in systematic reviews, because we did not statistically analyze outcome data as such. It should be borne in mind that none of the included articles had the aim of defining RTP or validating specific criteria to support the RTP decision. Another potential weakness is that not all of the studies defined hamstring injury or described the medical assessment. Thus, it cannot be excluded that study participants had other injuries causing posterior upper leg pain (such as referred pain or adductor-related injuries), injuries for which different RTP definitions and criteria might apply.

### Strengths and Weaknesses in Relation to Other Studies

As far as we know, this is the first review of the definitions and criteria for RTP after acute hamstring injury. In all the included articles, criteria for RTP focused on medical factors and thus results should be interpreted in the light of medical clearance for RTP. It has been suggested that modifiers of sport risk (e.g., type of sport, competitive level) and decisions (e.g., pressure, fear of litigation) should also be considered when determining readiness for RTP [1]. A practical decision-based RTP model of Creighton et al. guides us through three steps [1]. In step 1, medical factors such as age, injury history, psychological state, outcome of clinical tests, and imaging are evaluated. In step 2, sport-specific risk modifiers such as type, level of sport, and player position are evaluated. Finally in step 3, decision modifiers, such as timing in season, importance of match (e.g., final), external pressure, and financial conflicts of interest are considered. This means that the RTP decision should involve not only the medical doctor but also the player and other stakeholders [2].

To date, none of the RTP criteria have been validated with regard to the RTP decision after hamstring injury. Only a few studies included had a primary focus on investigating specific criteria for RTP [25, 26]. Reurink et al. described that at the time of RTP, 89 % of all clinically healed hamstring injuries still demonstrated increased signal intensity on MRI [26]. Tol et al. found that two-thirds of the players in their study group demonstrated a >10 % deficit on hamstring isokinetic testing [25]. They did not find differences in isokinetic strength parameters in players who sustained a re-injury [25]. The relationship between these deficits at the time of RTP and the risk of re-injury is not known. In addition, it should be considered that owing to the multifactorial condition and complexity of the hamstring injury, a more comprehensive assessment of the different risk factors should be included [59].

In a recent study, Mendiguchia et al. proposed a RTP algorithm that included criteria for progression through each rehabilitation phase, which could assist clinical decision making regarding RTP after hamstring injury [16]. This algorithm considers all risk factors that potentially affect hamstring injury risk and incorporates the current literature on biology of muscle injury and repair. A new active hamstring flexibility test, called the “H-test”, also seems a promising tool for assessing readiness for RTP after hamstring injury [38]. It is recommended that the test be performed at the end of rehabilitation, when other tests have indicated clinical recovery [38]. Askling et al. suggested that the risk of recurrent hamstring injury is significantly reduced if there are no signs of insecurity during the test [38]. These findings, if confirmed, may be an important first step to decreasing the high rates of re-injury and to optimizing RTP. Functional assessment peculiar to the given sport was also often suggested to support the RTP decision [25, 26, 37, 42, 43, 45–48, 50, 51, 53, 56]. However, a more comprehensive description of assessment parameters and limit values allowing therapists to authorize (or delay) RTP, such as ‘pre-injury-level’ or ‘asymmetry during running’, needs to be provided.

The lack of an unambiguous definition of and clear criteria for RTP after hamstring injury makes it difficult to compare and interpret study results. For example, the study by Hamid et al. [36] used lack of pain on direct palpation, no pain on hamstring contraction, symmetrical range of motion, and equal hamstring strength between affected and unaffected legs as criteria for RTP. In the study by Reurink et al., participants were required to complete, without experiencing symptoms, a functional criteria-based four-staged physiotherapy program, which included a final supervised sport-specific (outdoor) training phase, and to have a <10 % difference in isokinetic strength between the affected and unaffected legs [26]. Additionally, athletes were advised to have 5 days of additional team training before participation in a match [26]. The study of Askling et al. differed from these studies in that RTP was self-registered by the study participants, with participants reporting they could train/perform their sport again, regardless of whether they had symptoms [37]. While these articles have contributed to our knowledge of hamstring injury management, the differences in definitions and criteria for RTP will inevitably lead to a different time to RTP. Moreover, the actual timing of RTP probably reflects the success of treatment less than the choice of definition and criteria for RTP.

### Meaning of the Study: Possible Implications for Clinicians or Researchers

We found a lack of definitions of and criteria for RTP after acute hamstring injury in the literature, which could lead to different research outcomes. Recurrence rates, which can in part be explained by premature RTP, are still extremely high [8, 9]. Given the high recurrence rates and long rehabilitation for recurrent hamstring injuries, it is essential that clinicians have validated RTP criteria to support the RTP decision.

In the current literature, the definition of RTP after hamstring injury is based on the athlete reaching a pre-injury level of performance or being able to perform full sport activities and should be guided by medical advice. Clinical approval for RTP is commonly based on the athlete experiencing no pain, achieving a similar hamstring strength and flexibility as before injury, and performing properly on functional testing.

Establishing a definition and providing objective criteria for RTP after acute hamstring injury is essential for injury management, particularly the prevention of recurrent hamstring injuries. Therefore, future research should focus on achieving agreement on the definition of RTP and criteria to guide the RTP decision. Prospective studies are needed to validate these criteria and their correlation with successful RTP.

## Conclusion

Only half of the included studies provided some definition of RTP after hamstring injury, of which reaching the athlete’s pre-injury level of performance and being able to perform full sport activities were important elements. Numerous criteria are used to support the RTP decision, but none of these have been validated. Research is needed to reach a consensus on the definition of RTP and to provide validated RTP criteria to facilitate hamstring injury management and reduce hamstring injury recurrence.

## Electronic supplementary material

Below is the link to the electronic supplementary material.

Supplementary material 1 (DOCX 32 kb)
