# Supplementary material for: Return to Play After Hamstring Injuries: A Qualitative Systematic Review of Definitions and Criteria
Source: Sports Med. 2016 Jan 14;46:899–912. doi: 10.1007/s40279-015-0468-7 (PMC4887544; doi:10.1007/s40279-015-0468-7)
Supplement: Supplementary file 1 — Supplementary material 1 (DOCX 32 kb) [file 40279_2015_468_MOESM1_ESM.docx]

**Electronic Supplementary Material Appendix S1. Search strategies for all databases**

| ***Search database*** | ***Search string*** |
| --- | --- |
| *PubMed* | (hamstring[tiab] OR hamstrings[tiab] OR biceps femoris[tiab] OR semitendinosus[tiab] OR semitendinosus[tiab] OR semimembranosus[tiab] OR thigh[tiab] OR thighs[tiab] OR upper leg[tiab] OR upper legs[tiab]) AND (return to play[tiab] OR return to action[tiab] OR return to sport[tiab] OR return to sports[tiab] OR return to sporting activities[tiab] OR return to activity[tiab]OR return to competition[tiab] OR return to training[tiab] OR sports participation[tiab] OR return to level[tiab] OR sport participation[tiab] OR match fitness[tiab] OR training fitness[tiab] OR return to action[tiab] OR full fitness[tiab] OR repetitive injury[tiab] OR recurrent injuries[tiab] OR repetitive injuries[tiab] OR recurrent strain[tiab] OR repetitive strain[tiab]) OR recurrent strains[tiab] OR repetitive strains[tiab]) |
| *Embase - Medline* | (hamstring:ti,ab OR hamstrings:ti,ab OR “biceps femoris”:ti,ab OR semitendinosus:ti,ab OR semitendinosis:ti,ab OR semimembranosus:ti,ab OR thigh:ti,ab OR thighs:ti,ab OR “upper leg”:ti,ab OR “upper legs”:ti,ab) AND (“return to play”:ti,ab OR “return to action”:ti,ab OR “return to sport”:ti,ab OR “return to sports”:ti,ab OR “return to sporting activities”:ti,ab OR “return to activity”:ti,ab OR “return to competition”:ti,ab OR “return to training”:ti,ab OR “sports participation”:ti,ab OR “return to level”:ti,ab OR “sport participation”:ti,ab OR “match fitness”:ti,ab OR “training fitness”:ti,ab OR “return to action”:ti,ab OR “full fitness”:ti,ab OR “repetitive injury”:ti,ab OR “recurrent injuries”:ti,ab OR “repetitive injuries”:ti,ab OR “recurrent strain”:ti,ab OR “repetitive strain”:ti,ab OR “recurrent strains”:ti,ab OR “repetitive strains”:ti,ab) |
| *CINAHL* | hamstring OR hamstrings OR biceps femoris OR semitendinosus OR semitendinosus OR semimembranosus OR thigh OR thighs OR upper leg OR upper legs AND return to play OR return to action OR return to sport OR return to sports OR return to sporting activities OR return to activity OR return to competition OR return to training OR sports participation OR return to level OR sport participation OR match fitness OR training fitness OR return to action OR full fitness OR repetitive injury OR recurrent injuries OR repetitive injuries OR recurrent strain OR repetitive strain OR recurrent strains OR repetitive strains |
| *PEDro* | hamstring AND return |
| *Cohrane Library* | (hamstring or hamstrings or “biceps femoris” or semitendinosus or semitendinosus or semimembranosus or thigh or thighs or “upper leg” or “upper legs”) and (“return to play” or “return to action” or “return to sport” or “return to sports” or “return to sporting activities” or “return to activity” or “return to competition” or “return to training” or “sports participation” or “return to level” or “sport participation” or “match fitness” or “training fitness” or “return to action” or “full fitness” or “repetitive injury” or “recurrent injuries” or “repetitive injuries” or “recurrent strain” or “repetitive strain” or “recurrent strains” or “repetitive strains”) |
| *Scopus* | (TITLE-ABS-KEY((hamstring or hamstrings or "biceps femoris" or thigh or thighs or "upper leg" or "upper legs"))) AND (TITLE-ABS-KEY(("return to play" or "return to sport" or "return to sports" or "return to competition" or "sports participation" or "full fitness" or "training fitness" or "match fitness"))) |
| *SportDiscus* | hamstring OR hamstrings OR biceps femoris OR semitendinosus OR semitendinosus OR semimembranosus OR thigh OR thighs OR upper leg OR upper legs AND return to play OR return to action OR return to sport OR return to sports OR return to sporting activities OR return to activity OR return to competition OR return to training OR sports participation OR return to level OR sport participation OR match fitness OR training fitness OR return to action OR full fitness OR repetitive injury OR recurrent injuries OR repetitive injuries OR recurrent strain OR repetitive strain OR recurrent strains OR repetitive strains |
